# Single-dose acarbose decreased glucose-dependent insulinotropic peptide and glucagon levels in Chinese patients with newly diagnosed type 2 diabetes mellitus after a mixed meal

**DOI:** 10.1186/s12902-016-0133-7

**Published:** 2016-09-29

**Authors:** Zhong Chen, Xiaoying Fu, Jian Kuang, Ju Chen, Hongmei Chen, Jianhao Pei, Huazhang Yang

**Affiliations:** Department of Endocrinology, Guangdong General Hospital/Guangdong Academy of Medical Sciences, Guangzhou, 510080 Guangdong China

**Keywords:** Acarbose, Glucagon-like peptide-1, Glucose-dependent insulinotropic peptide, Glucagon, Type 2 diabetes mellitus

## Abstract

**Background:**

Acarbose slows down the intestinal absorption of carbohydrates, but its effects on the secretion of incretins are still poorly known. This study aimed to examine the effects of single-dose acarbose on the secretion of incretins in patients with newly diagnosed type 2 diabetes mellitus (T2DM).

**Methods:**

In this pilot study, twenty-three patients diagnosed with T2DM were randomly assigned to the oral glucose tolerance test (OGTT) group (*n* = 11) and the mixed meal test (MMT) group (*n* = 12). Fourteen subjects with normal OGTT were included as controls. Plasma glucose, insulin, glucagon, glucagon-like peptide-1 (GLP-1), and glucose-dependent insulinotropic peptide (GIP) were measured at 0 (fasting), 15, 30, 60, 90, and 120 min after nutrient load. A week later, controls underwent MMT, the OGTT group underwent OGTT receiving 100 mg acarbose, and the MMT group underwent MMT receiving 100 mg acarbose. The same blood markers were measured again.

**Results:**

No significant difference was observed in the OGTT group before and after administering acarbose. In the MMT group, postprandial levels of glucose (*P* < 0.01), insulin (*P* < 0.01), glucagon at 15 min (*P* < 0.05), glucagon area under the curve (AUC) (*P* < 0.05), GIP levels at 30 min (*P* < 0.05), and GIP AUC (*P* < 0.05) were decreased after receiving acarbose with a mixed meal, but GLP-1 levels and GLP-1 AUC did not change.

**Conclusions:**

Single-dose acarbose could reduce the secretion of GIP and glucagon after a mixed meal in patients with newly diagnosed T2DM. The influence of acarbose on incretin levels could be related to the types of carbohydrate being consumed.

**Trial registration:**

This study was registered with the Chinese Clinical Trial Registry (Registration Number: ChiCTR-TRC-14004260, Date of Registration: 2014-01-19).

## Background

Considerable evidence suggests that incretin dysfunction accompanies the development of type 2 diabetes mellitus (T2DM). Incretins are estimated to account for approximately 50–70 % of the total insulin secreted after oral glucose administration [1–3]. The most important incretins are glucagon-like peptide-1 (GLP-1) and glucose-dependent insulinotropic peptide (GIP). GLP-1 is secreted from intestinal L cells when triggered by ingested nutrients, while GIP is released from intestinal K cells. Studies have demonstrated that an impaired secretion of incretins in patients with T2DM is characterized by a decreased secretion of GLP-1 after a mixed meal, resulting in the dysfunction of insulinotropic actions, or a total loss of GIP effect even with normal levels [4]. However, many factors may affect incretin secretion including meal composition, obesity, insulin resistance, glucose intolerance, gastric-emptying, and glucose-lowering drugs [5], and their exact relationships are still poorly known.

Acarbose, an α-glucosidase inhibitor, is a polysaccharide that could be used as a hypoglycemic drug since it slows down the intestinal absorption of carbohydrates [6]. Acarbose binds to the α-glycosidase and thus inhibits food polysaccharides or disaccharides from being decomposed into monosaccharide and subsequently absorbed into the bloodstream [6]. Therefore, acarbose can decrease postprandial blood glucose from increasing through decreased intestinal absorption [6]. Since acarbose do not bind covalently to α-glycosidase but competitively, it has to be consumed at the same time as the meals. Acarbose is seldom absorbed into the bloodstream (<5 %), and there is no pharmacokinetic issues such as effective drug concentration in the blood. Acarbose is excreted through the feces [6]. Studies have shown increased postprandial levels of GLP-1 in normal subjects receiving acarbose [7, 8], but this effect was not observed in patients with T2DM [9]. A previous study suggested that a 24-week treatment with acarbose could stimulate the secretion of GLP-1 [10]. However, it was difficult to identify that the effect on the incretins was due to the long-term improvement of glucotoxicity or the intrinsic action of acarbose.

There are profound differences in the dietary patterns between the Chinese and Western populations [11, 12], and these differences could confound the associations observed in Western populations when food intake is involved. We hypothesized that acarbose affected the secretion of incretins in patients with newly diagnosed T2DM. Therefore, the present study aimed to identify whether a single dose of acarbose had the potential to affect incretin levels beyond its glycemic effect in drug-naive Chinese patients newly diagnosed with T2DM.

## Methods

### Study subjects

In this pilot study, patients newly diagnosed with T2DM and healthy volunteers recruited from the endocrinology outpatient clinic of the Guangdong General Hospital from January 2009 to June 2010.

For patients, the inclusion criteria were: 1) newly diagnosed T2DM within 3 months according to the WHO diabetes diagnostic criteria of 1999 [13]; (2) aged 30–70 years; and (3) fasting plasma glucose (FPG) >7 mmol/L and ≤9 mmol/L and/or 2-h plasma glucose >11.1 and ≤16.6 mmol/L, Hb_A1c_ ≤9 %, and body mass index (BMI) ≥18 kg/m^2^.

Fourteen subjects with normal oral glucose tolerance test (OGTT) were included as controls. The inclusion criteria for the control group were: 1) age 30–70 years; and 2) OGTT FPG <5.6 mmol/L and 2-h plasma glucose <7.8 mmol/L.

For all participants, exclusion criteria were: 1) treatment with any antidiabetic drugs or drugs that affect insulin sensitivity, plasma glucose, or carbohydrate metabolism (glucocorticoids, diuretics, or beta-blocker); 2) history of gastrointestinal disease or surgery; 3) history of gastrointestinal disease or received treatment that affect gastroenteric movement; 4) history of nephropathy, hepatopathy, or cardiovascular disease; 5) history of diseases associated with flatulence such as Roemheld syndrome, enterocele, intestinal obstruction, or ulcer; 6) subjects having moderate to severe renal or hepatic insufficiency (ALT >2.5-fold the upper normal limit (ULN), and plasma creatinine > ULN); or 7) pregnant women.

The study was approved by the local medical ethics committee. All participants provided a written informed consent. This study was registered with the Chinese Clinical Trial Registry (ChiCTR-TRC-14004260, http://www.chictr.org.cn).

### Study design and intervention

**Fig. 1:**
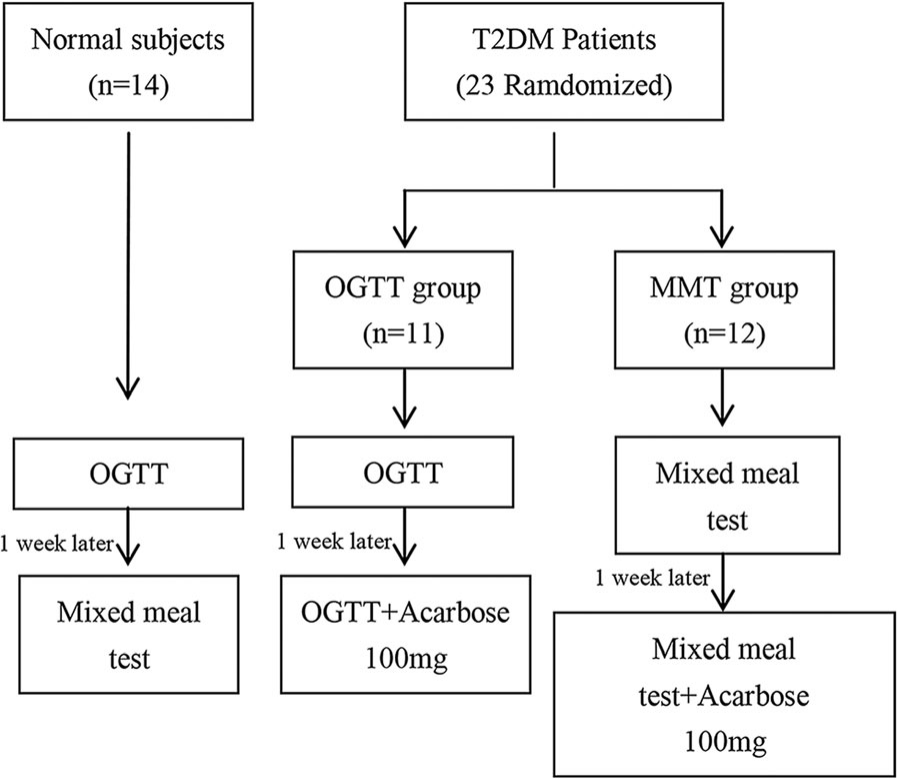
Study flowchart

Twenty-three patients newly diagnosed with T2DM and meeting the inclusion criteria were randomly divided into two groups using a random number table (Fig. 1): the OGTT group and the mixed meal test (MMT) group. The control and OGTT groups received 75 g of anhydrous glucose orally, while the MMT group received a mixed meal with a total calorie input of 370 kcal (carbohydrates 74 %, proteins 7 %, and fat 19 %). A week later, the control group underwent MMT, and the other two groups received 100 mg of acarbose orally (Glucobay®; Bayer AG, Leverkusen, Germany) along with the same nutrient intake as the first time.

### Laboratory measurements

Blood samples were collected in the morning after a 12-h overnight fast. Samples were drawn into chilled tubes containing EDTA and aprotinin for the measurement of plasma glucose, insulin, glucagon, and GIP at 0 (fasting), 15, 30, 60, 90, and 120 min after the nutrient load. Blood samples for measuring GLP-1 were collected into chilled tubes containing EDTA and DPP-4 inhibitor (Linco Cat# DPP4, 10 μL of DPP-4 inhibitor per milliliter of blood) within 30 s. After centrifugation, plasma samples were kept at −70 °C until assayed.

Plasma GLP-1 (Cat No. GLP-1A-35HK, Linco Research, Inc., St. Charles, MO, USA) [14, 15], GIP (Cat. No. RKC-027-02, Phoenix Pharmaceuticals, Inc., Burlingame, CA, USA) [16], and glucagon (Cat No. GL-32 k, Linco Research, Inc., St. Charles, MO, USA) [17] were measured using commercially available radioimmunoassay kits, according to the manufacturers’ instructions. The intra-assay coefficient of variation (CV) for GLP-1 was 21.2–30.3 %, and the inter-assay CV was 12–43 %. The intra-assay CV for GIP was <10 %, while the inter-assay CV was <15 %. The intra-assay CV for glucagon was 4.0–6.8 % and the inter-assay CV was 7.3–13.5 %.

### Endpoints

The primary endpoint was the differences in the secretion of incretins after a single dose of acarbose. The secondary endpoints included the effect of acarbose on glucagon and the differences between newly diagnosed patients with T2DM and healthy subjects in the secretion of incretins and glucagon, and the changes in plasma glucose and insulin after acarbose in T2DM.

### Statistical analysis

Normally distributed continuous data are expressed as mean ± standard deviation. Non-normally distributed continuous data were log-transformed to normalize their distribution. Data were compared using ANOVA and the Tukey’s post hoc test. Differences in categorical variables were tested using the chi-square test. Statistical analysis was performed using SPSS 13.0 (SPSS, Inc., Chicago, IL, USA). Two-sided *P*-values <0.05 were considered significant.

## Results

### Characteristics of the participants

Twenty-three patients newly diagnosed with T2DM and 14 healthy volunteers were recruited (Fig. 1). The characteristics of the participants are shown in Table 1. Significant differences in age, BMI, and waist circumference were observed between the controls and patients with T2DM (*P* < 0.05). Hb_A1c_, systolic blood pressure, total cholesterol, and low-density lipoprotein cholesterol were significantly higher in patients with T2DM than in healthy controls (*P* < 0.05). There was no significant difference for all other characteristics between the two groups (*P* > 0.05).

**Table 1:** Characteristics of the participants

Characteristic	Control group	OGTT group	MMT group
Number (male/female)	14 (7/7)	11 (5/6)	12 (9/3)
Age (year)	32.21 ± 3.13	54.45 ± 3.46*	56.75 ± 2.10*
BMI (kg/m^2^)	21.56 ± 0.63	25.02 ± 1.06*	25.52 ± 1.11*
WHR	0.81 ± 0.02	0.88 ± 0.02*	0.90 ± 0.02*
HB_A1c_ (%)	5.6 ± 0.1	7.3 ± 0.4*	7.7 ± 0.4*
HBA1c (mmol/mL)	37 ± 1	56 ± 4*	61 ± 4*
SBP (mmHg)	112.21 ± 3.19	128.55 ± 3.18*	127.17 ± 4.43*
DBP (mmHg)	71.21 ± 2.64	78.45 ± 1.76*	74.33 ± 1.38
TC (mmol/L)	4.52 ± 0.17	5.60 ± 0.31*	5.39 ± 0.23*
TG (mmol/L)	0.88 ± 0.07	2.31 ± 0.58*	1.69 ± 0.40*
HDL-C (mmol/L)	1.38 ± 0.08	1.37 ± 0.12	1.40 ± 0.13
LDL-C (mmol/L)	2.07 ± 0.12	2.65 ± 0.16*	2.65 ± 0.22*
ALT (U/L)	23.17 ± 3.14	32.89 ± 7.55	26.00 ± 2.74
BUN (mmol/L)	4.38 ± 0.29	5.60 ± 0.38*	4.956 ± 0.39
Cr (μmol/L)	69.82 ± 6.40	78.22 ± 5.15	83.58 ± 4.69
Uric acid (μmol/L)	317.57 ± 15.13	353.25 ± 15.61	333.08 ± 17.57

*BMI* Body mass index, *WHR* waist-to-hip ratio, *SBP* systolic blood pressure, *DBP* diastolic blood pressure, *TC* total cholesterol, *TG* triglyceride, *HDL-C* high-density lipoprotein cholesterol, *LDL-C* low-density lipoprotein cholesterol, *ALT* alanine aminotransferase, *BUN* blood urea nitrogen, *Cr* creatinine

*P < 0.05 vs. controls

△*P* < 0.05 vs. the OGTT group

### Plasma glucose, insulin, and glucagon before and after acarbose

Before administering acarbose, plasma glucose in the OGTT group was higher, and insulin levels at 15 min (*P* < 0.01) and 30 min (*P* < 0.05) were lower than in the control group after receiving an oral 75 g glucose load. However, no difference was found in glucagon levels between the groups. When given a mixed meal, the MMT group also presented high levels of plasma glucose and low levels of insulin at 15 and 30 min (*P* < 0.05 vs. controls), but glucagon levels in the MMT group at 30 and 90 min were increased compared with the control group (*P* < 0.05) (Table 2).

**Table 2:** Comparison of plasma glucose, insulin, glucagon, GLP-1, and GIP before and after the administration of acarbose

	75-g glucose test			Mixed meal test		
Parameters	Control group (*N* = 14)	OGTT group Before^§^ (*N* = 11)	OGTT group After^☆^ (*N* = 11)	Control group (*N* = 14)	MMT group Before (*N* = 12)	MMT group After (*N* = 12)
Plasma glucose(mmol/L)						
0 min	4.77 ± 0.13	6.95 ± 0.56**	6.67 ± 0.51	4.73 ± 0.11	6.53 ± 0.36△△	6.49 ± 0.30
15 min	7.29 ± 0.25	8.88 ± 0.59*	9.16 ± 0.57	6.46 ± 0.21^#^	7.69 ± 0.44△	6.72 ± 0.31^▲^
30 min	8.47 ± 0.42	11.29 ± 0.60**	11.23 ± 0.66	7.59 ± 0.22	9.92 ± 0.47△△	7.39 ± 0.47^▲▲^
60 min	7.74 ± 0.50	13.66 ± 0.83**	12.89 ± 0.78	6.62 ± 0.38	13.22 ± 0.66△△	8.70 ± 0.48^▲▲^
90 min	6.17 ± 0.32	13.80 ± 0.95**	13.43 ± 1.03	5.17 ± 0.30	13.11 ± 0.87△△	9.45 ± 0.61^▲▲^
120 min	5.58 ± 0.18	12.95 ± 1.06**	12.39 ± 1.16	5.36 ± 0.26	11.65 ± 1.03△△	9.11 ± 0.72^▲▲^
Insulin (pmol/L)						
0 min	44.96 ± 4.86	62.66 ± 9.27	63.15 ± 9.63	47.32 ± 7.43	61.73 ± 10.03	40.08 ± 18.76
15 min	437.26 ± 70.09	196.51 ± 70.16**	194.43 ± 41.90	419.56 ± 114.74	118.39 ± 14.66△△	102.41 ± 24.43
30 min	586.97 ± 65.15	330.84 ± 76.93*	254.87 ± 41.78	566.20 ± 114.52	213.46 ± 50.03△△	128.99 ± 28.18^▲▲^
60 min	646.02 ± 136.59	448.28 ± 72.11	395.81 ± 45.47	497.23 ± 123.59	321.36 ± 71.54	178.20 ± 43.16^▲▲^
90 min	492.54 ± 91.56	563.69 ± 95.32	501.14 ± 65.87	377.62 ± 104.38	387.32 ± 79.81	229.93 ± 67.12^▲▲^
120 min	288.95 ± 47.35	453.19 ± 63.14	480.14 ± 72.53	248.76 ± 83.14	353.76 ± 65.63△	196.95 ± 48.54^▲▲^
Glucagon (pmol/L)						
0 min	70.24 ± 5.94	74.92 ± 8.21	70.05 ± 6.58	74.24 ± 4.83	80.54 ± 5.77	61.62 ± 6.18^▲▲^
15 min	75.72 ± 8.68	78.52 ± 8.37	81.32 ± 11.82	92.13 ± 7.47	99.40 ± 8.40	77.88 ± 9.07^▲^
30 min	69.41 ± 6.97	83.81 ± 6.48	70.83 ± 5.63	70.82 ± 4.35	94.35 ± 8.82△	88.34 ± 8.28
60 min	69.46 ± 7.10	63.09 ± 5.39	61.28 ± 7.00	62.31 ± 3.87	79.23 ± 7.47	77.06 ± 6.41
90 min	59.01 ± 5.61	64.00 ± 8.29	51.41 ± 6.32	63.80 ± 3.72	82.20 ± 8.10△	73.31 ± 9.72
120 min	59.81 ± 4.24	58.38 ± 6.90	55.25 ± 6.33	74.24 ± 4.83	63.20 ± 4.34	60.14 ± 7.74
GLP-1 (pmol/L)						
0 min	37.02 ± 3.95	36.46 ± 4.84	44.53 ± 6.73	35.90 ± 3.08	30.54 ± 4.71	41.87 ± 6.80
15 min	39.79 ± 5.79	33.51 ± 5.76	42.29 ± 5.62	35.85 ± 4.49	43.90 ± 6.58	36.43 ± 7.72
30 min	29.89 ± 3.04	32.93 ± 6.06	35.29 ± 4.22	33.66 ± 3.91	37.48 ± 5.21	35.44 ± 5.83
60 min	33.18 ± 3.72	35.19 ± 4.78	35.82 ± 6.88	30.20 ± 4.09	35.44 ± 4.80	43.42 ± 6.58
90 min	29.34 ± 3.17	37.05 ± 5.60	39.81 ± 7.89	28.45 ± 3.07	39.12 ± 5.01	35.33 ± 4.98
120 min	31.20 ± 5.17	30.73 ± 4.22	29.42 ± 5.20	31.51 ± 3.37	41.48 ± 5.74	32.58 ± 5.43
GIP (pmol/L)						
0 min	31.85 ± 3.61	31.94 ± 12.18	43.53 ± 13.96	37.33 ± 5.93	34.36 ± 3.67	33.86 ± 8.82
15 min	46.19 ± 4.97	82.50 ± 26.80	55.12 ± 13.82	78.87 ± 15.89^#^	58.42 ± 19.96	31.27 ± 6.20
30 min	41.39 ± 5.89	52.65 ± 22.53	56.66 ± 17.46	68.23 ± 16.42	76.52 ± 16.98	41.99 ± 8.97^▲^
60 min	41.30 ± 4.87	76.49 ± 13.61*	72.78 ± 19.30	42.36 ± 8.21	78.95 ± 25.37	59.34 ± 13.92
90 min	50.07 ± 10.14	59.62 ± 18.67	94.76 ± 25.21	61.43 ± 14.92	88.63 ± 25.35	63.57 ± 18.41
120 min	52.23 ± 9.94	65.50 ± 21.84	57.68 ± 9.81	50.39 ± 8.80	72.86 ± 20.73	33.46 ± 4.32

*P < 0.05 OGTT group vs. controls before the administration of acarbose

**P < 0.01 OGTT group vs. controls before the administration of acarbose

^#^ *P* < 0.05 *P* < 0.05 MTT vs. OGTT in conrols

△*P* < 0.05 MMT group vs. controls group before the administration of acarbose

△△*P* < 0.01 MMT group vs. controls before the administration of acarbose

^▲^ *P* < 0.05 MMT group before and after the administration of acarbose

^▲▲^ *P* < 0.01 MMT group before and after the administration of acarbose

^§^Before the administration of acarbose

^☆^: After the administration of acarbose

Administering acarbose did not alter the levels of plasma glucose, insulin, and glucagon in the OGTT group at any time point, but plasma glucose was decreased in the MMT group after receiving acarbose. Insulin levels were decreased significantly at 30, 60, 90, and 120 min after administering acarbose in the MMT group, and glucagon was also decreased at 15 min (Table 2).

### Levels of GLP-1 and GIP before and after administering acarbose

Without acarbose, no difference was found in GLP-1 levels between the control and OGTT groups after the 75-g glucose load, but GIP levels were higher at 60 min in the OGTT group compared with the control group (*P* < 0.05). When given a mixed meal, the levels of GLP-1 and GIP in the MMT group did not differ from the control group (Table 2).

Acarbose did not induce any difference in the levels of GLP-1 and GIP in the OGTT group. The levels of GLP-1 in the MMT group did not change after single-dose acarbose, but the GIP levels of the MMT group at 30 min were decreased significantly after acarbose (Table 2).

### Area under the curve of plasma glucose, insulin, glucagon, GLP-1, and GIP before and after acarbose

**Fig. 2:**
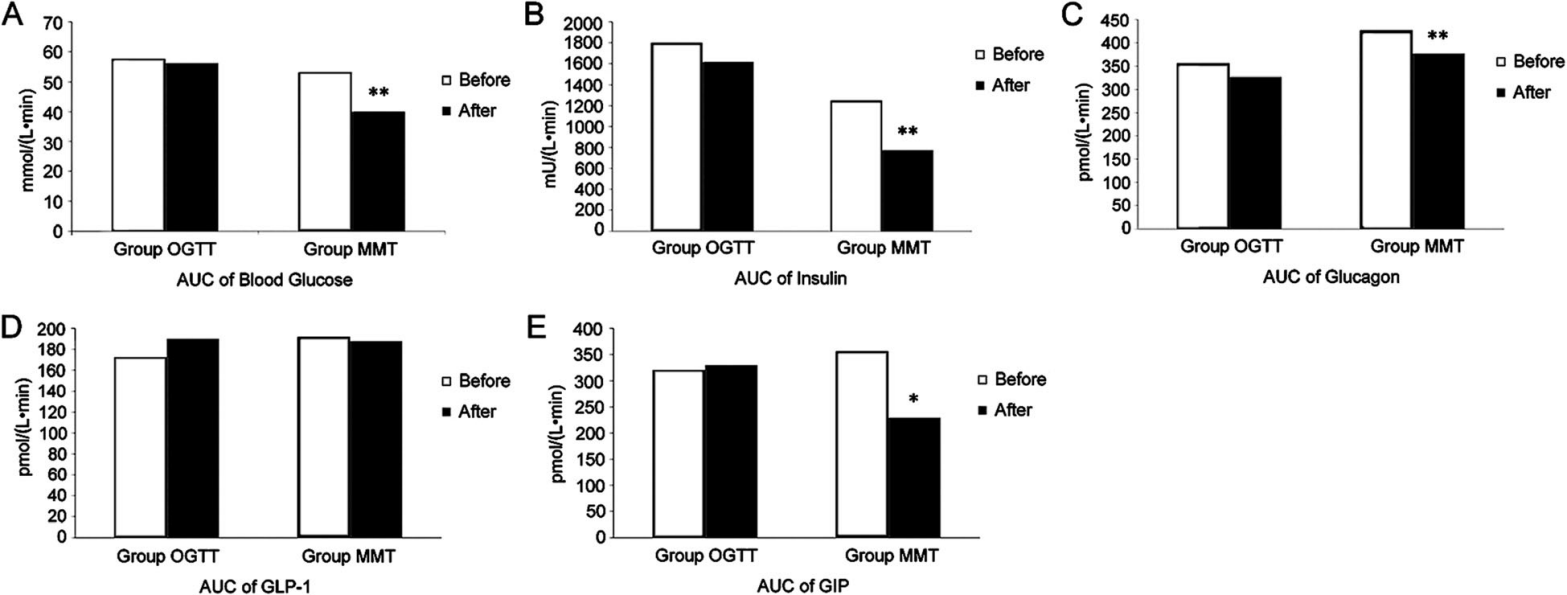
Effect of acarbose on an oral glucose tolerance test before and after acarbose administration in newly diagnosed patients with type 2 diabetes. **a** Area under the curve (AUC) for plasma glucose. **b** AUC for insulin levels. **c** AUC of glucagon. **d** AUC of GLP-1. **e** AUC of GIP

Before acarbose, the area under the curve (AUC) of plasma glucose in the OGTT group was markedly higher than in the control group, but no difference was found in the AUCs of insulin, glucagon, GLP-1, and GIP between the two groups. When given a mixed meal, the AUCs of plasma glucose (*P* < 0.01) and glucagon (*P* < 0.05) increased in the MMT group compared with the control group, but the AUC of insulin was not different neither were the AUCs of GLP-1 and GIP (Fig. 2).

Administering acarbose made no difference in the AUCs of plasma glucose, insulin, glucagon, GLP-1, and GIP in the OGTT group. The AUCs of plasma glucose, insulin, and glucagon in the MMT group were significantly different before and after administering acarbose (*P* < 0.01), and so was the AUC of GIP (*P* < 0.05). However, the AUC of GLP-1 showed no difference with the use of acarbose.

## Discussion

The aim of the present study was to try to examine the intrinsic effects of acarbose on the secretion of incretins in drug-naive patients with newly diagnosed T2DM. Results showed that no significant difference was observed in the OGTT group before and after administering acarbose. In the MMT group, postprandial levels of plasma glucose, insulin, glucagon at 15 min, glucagon AUC, GIP levels at 30 min, and GIP AUC were decreased after receiving acarbose with a mixed meal, but GLP-1 levels and GLP-1 AUC did not change. These results suggest that the influence of acarbose on incretin levels is related to the types of carbohydrate being consumed in patients with T2DM.

Inhibitors of α-glucosidase (such as acarbose) are known to delay the intestinal hydrolysis of oligo- and disaccharides, mainly in the upper half of the small intestine, by binding competitively and in a dose-dependent manner to α-glucosidase, reducing their intestinal absorption, but acarbose has no direct effect on the absorption of glucose [18]. As a result, after taking 75 g glucose, the OGTT group in this study did not present significant changes in the levels of plasma glucose with the administration of acarbose. Furthermore, the results showed no difference in the levels of incretins and insulin in the OGTT group after administering acarbose, indicating that acarbose had no direct effect on the secretion of incretins from L and K cells after the 75 g glucose load.

In the present study, after acarbose, the MMT group did not present significant difference in GLP-1, but the levels at 30 min and the AUC of GIP were decreased significantly. These findings are inconsistent with those of other studies. Indeed, GLP-1 secretion is dependent on both the size and the nutrient composition of the meal [4]. Vilsboll et al. [19] demonstrated that the incretin responses were significantly higher in the group receiving a large meal compared with those receiving a small meal in lean patients with type 1 diabetes mellitus and obese patients with T2DM (520 kcal vs. 260 kcal). Meal sizes of 400–550 kcal have failed to demonstrate that acarbose could enhance GLP-1 secretion [14, 20]. A previous study has shown that miglitol, a first-generation α-glucosidase inhibitor, induced an enhanced GLP-1 release with an ordinary meal of 720 kcal in obese women with T2DM [21]. In another study using oral sucrose, patients with poorly controlled T2DM also showed a prolonged and enhanced secretion of GLP-1 after administration of acarbose [22]. In the present study, the meal size was 370 kcal and was designed to represent the characteristic Chinese meal. It included 74 % carbohydrates, 7 % protein, and 19 % fat [23, 24], which might not be sufficient to stimulate the secretion of GLP-1 even with the administration of acarbose. However, factors such as the inclusion criteria of subjects (duration of diabetes, severity of diabetes complications, combined medications, and the degree of obesity and insulin resistance) might influence incretin secretion. In the present study, patients were newly diagnosed with T2DM and were slightly overweight (BMI of 25.5 ± 1.1 kg/m^2^), and they had no history of antidiabetic drugs or chronic complications of diabetes. Therefore, GLP-1 might not yet be impaired severely in these patients. In addition, subjects in this study received a single dose of acarbose, which was different from other studies. Indeed, Zheng et al. [10] reported that a 24-week treatment with acarbose significantly increased the levels of GLP-1 in newly diagnosed T2DM patients. The β cells function and insulin sensitivity can be improved by the elimination of glucotoxicity, which can affect the secretion of incretins directly and indirectly [5]. Glycemic control after the 24-week administration of acarbose may contribute in part to the restoration of the GLP-1 secretion defect. However, no significant changes were found in GLP-1 levels with the single dose of acarbose.

Recent studies of inhibitors of α-glucosidase (acarbose and miglitol) in patients with T2DM have indicated that these drugs can reduce the levels of GIP [20, 25, 26]. GIP is released from K cells in the duodenum, which can be stimulated through the absorption of carbohydrates and fat [27]. Studies have shown that the secretion of GIP increases significantly after the excessive ingestion of nutrients, indicating that GIP plays an important role in the development of obesity and insulin resistance induced by a high-calorie diet [28]. The present study showed that the GIP and glucagon levels decreased after a mixed meal in patients with new diagnosed T2DM by treatment with single dose acarbose.

It is known that the secretion and activation of incretins are impaired in patients with T2DM [5], and this dysfunction can be observed even in the early stage of glucose metabolism disorders [29]. Incretin dysfunction is not necessarily a characteristic unique to T2DM because several studies reported no GLP-1 deficiency in the early stage of abnormal glucose tolerance, suggesting that incretin deficiency could be the consequence of T2DM development [30, 31]. In addition, patients with long duration of diabetes and poor response of glucagon to glucose stimulation may present GLP-1 dysfunction [5]. A previous study showed that acarbose decreased GLP-1 levels in patients with T2DM [10]. In the present study, subjects were newly diagnosed with diabetes and had a mean Hb_A1c_ of 7.3 ± 0.4 % and a mean BMI of about 25 kg/m^2^, which may explain why no difference was found in GLP-1 levels between healthy subjects and patients with T2DM. A possible explanation is the fact that the effect of meals on GLP-1 secretion is negligible in East Asians, as well as GLP-1 secretion after OGTT [32]. Additional study is necessary to address this issue.

On the other hand, GIP levels were increased significantly at 60 min during OGTT in patients with T2DM compared with controls. The present study also showed that insulin levels in the early phase were reduced while GIP was relatively high, suggesting that the biological action of GIP on stimulating glucose-dependent insulin secretion was impaired. Such phenomenon was demonstrated in a previous study of patients with T2DM, in whom GIP levels were normal or increased but the insulinotropic response was diminished substantially [5]. As for the present study, the differences in GIP between the controls and patients with T2DM may suggest that the early changes in T2DM development may occur earlier than the changes in GLP-1 levels.

OGTT is a load of pure glucose. Since acarbose delays the hydrolysis of polysaccharides and disaccharides but has no effect on the direct absorption of glucose, acarbose had no effect on the OGTT parameters [18]. On the other hand, since the mixed meal contained polysaccharides and disaccharides, acarbose delayed their hydrolysis, resulting in changes in incretins. These results are supported by a previous study that showed some effects of a single dose acarbose after a meal tolerance test [33].

This study has several limitations. First, the number of subjects was probably not sufficient to provide definitive evidence of the effect of acarbose on incretins. Indeed, the effects of acarbose on incretins may be subtle, leading to differences that cannot be detected using a small sample size. In addition, variations in the methods used to measure incretin levels may confound the detection of differences, particularly with a small sample size. Secondly, the controls were not perfectly matched to the patients. Finally, the generalisability of these results may be limited by the particularities of the T2DM observed in Chinese [32]. Indeed, T2DM in Chinese is characterized by a rapid increase in prevalence due to the Westernized lifestyle changes, as well as specific genetic characteristics that result in low insulin secretory capacity in East Asians [32]. Further study is still necessary to determine adequately the effects of acarbose on the metabolism of incretins.

## Conclusions

A single dose of acarbose in drug-naive patients newly diagnosed with T2DM was found to decrease GIP and glucagon only in MMT rather than OGTT, indicating that these hormones might be influenced by acarbose through the delayed absorption of nutrients. The influence of acarbose on incretin levels could be related to the types of carbohydrate intake in patients with T2DM.

## Abbreviations

BMI: Body mass index
CV: Coefficient of variation
FPG: Fasting plasma glucose
GIP: Glucose-dependent insulinotropic peptide
GLP-1: Glucagon-like peptide-1
MMT: Mixed meal test
OGTT: Oral glucose tolerance test
T2DM: Type 2 diabetes mellitus
ULN: Upper normal limit
